# Analysis of differential photoplethysmography signal patterns in apnea and hypopnea

**DOI:** 10.1088/1361-6579/ae3ef0

**Published:** 2026-02-11

**Authors:** Márton Áron Goda, Arie Oksenberg, Ali Azarbarzin, Joachim A Behar

**Affiliations:** 1 Faculty of Biomedical Engineering, Technion Institute of Technology, Technion-IIT, Haifa 32000, Israel; 2 Pázmány Péter Catholic University Faculty of Information Technology and Bionics, Budapest, Práter u. 50/A, 1083, Hungary; 3 Departments of Medicine and Neurology, Harvard Medical School/Brigham and Women’s Hospital, Boston, MA 02115, United States of America

**Keywords:** photoplethysmography, sleep, respiratory events, body posture, machine learning

## Abstract

*Objective.* Photoplethysmography, a non-invasive optical technique that measures changes in blood volume in the microvascular bed of tissue, offers a promising approach for monitoring physiological changes during sleep. This study evaluates differential photoplethysmography signal patterns that can distinguish between apneas vs hypopneas, which are key features of sleep-related breathing disorders. *Approach.* We analyzed data from 263 severe (apnea hypopnea index ⩾30) obstructive sleep apnea patients, using recordings from the Multi-Ethnic Study of Atherosclerosis. Over 57 000 respiratory events occurring during stage N2 sleep were included. A machine learning model was trained on 89 features derived from the photoplethysmography signal, using the pyPPG toolbox, to classify: apneas vs hypopneas in the supine and lateral sleep posture, and posture-specific differences for each respiratory event type. *Main results.* Results showed that photoplethysmography signal characteristics significantly differed between apneas vs hypopneas. The model achieved an area under the receiver operation characteristic curve of 0.80 in the lateral posture and 0.83 in the supine posture. However, classification performance was low when distinguishing between apneas and hypopneas in the lateral vs the supine position with an area under the receiver operation characteristic curve of 0.62 for apneas and 0.64 for hypopneas. The discriminative signal features were consistent across different periods of the night. *Significance.* These findings indicate that photoplethysmography can detect meaningful differences in sleep-related breathing events and support its potential as a foundation for wearable diagnostic and monitoring tools that are personalized, accessible, and cost-effective.

## 1. Introduction

Obstructive sleep apnea (OSA) is a highly prevalent sleep disorder characterized by repeated episodes of complete (apnea) or partial (hypopnea) airflow cessation during sleep, leading to intermittent hypoxia and sleep fragmentation (Kapur *et al* 2017). Affecting an estimated 900 million adults worldwide, its prevalence and severity vary widely across populations (Benjafield *et al* 2019). OSA severity is classified using the apnea-hypopnea index (AHI), with diagnosis typically based on AHI values and associated symptoms or comorbidities. Apneas cause more severe oxygen desaturation and arousals than hypopneas, and longer event durations exacerbate these effects. These physiological differences suggest that apneas and hypopneas may carry distinct diagnostic and prognostic value. Both event types affect cardiovascular parameters, causing drops in blood pressure, heart rate, and oxygen saturation, followed by rapid increases and arousals. Notably, photoplethysmography (PPG) signals show increased amplitude during apneas (due to vasodilation) and decreased amplitude post-event (due to sympathetic-driven vasoconstriction), highlighting their potential for detecting these events (Hirotsu *et al* 2020).

PPG is a non-invasive optical technique that measures blood volume changes in the microvascular bed of tissue (Charlton *et al* 2022,2023). Wearable devices such as smartwatches and fitness trackers, which use PPG technology, have become widely popular, with millions of people using them for seamless and continuous health monitoring (Natarajan *et al* 2020). PPG sensor captures the arterial pulse wave created during the cardiac cycle, offering insights into various physiological systems, including the heart, blood vessels, microvasculature, autonomic nervous system, and respiratory system ((Nitzan *et al* 1998), (Meredith *et al* 2012)). PPG signals are obtained using a light source (e.g. LED) and a light sensor (e.g. photodiode) that measure light transmitted through or reflected from the skin. PPG’s applications extend beyond traditional heart rate and oxygen saturation monitoring to emerging uses such as blood pressure estimation (Mousavi *et al* 2019), atrial fibrillation detection (Pereira *et al* 2020), and assessment of sleep patterns (Lazazzera *et al* 2021), mental health (Loh *et al* 2022), and peripheral arterial disease (Allen *et al* 2021). Its ease of use, affordability, and unobtrusive nature make it widely adopted, though motion artifacts and other factors can impact signal quality.

PPG plays a pivotal role in modern sleep analysis due to its ability to track key physiological metrics such as heart rate variability, respiratory patterns, and blood oxygen saturation (Nemcova *et al* 2020). By analyzing variations in the PPG signal during sleep, wearable devices and research studies can identify different sleep stages, including light, deep, and REM sleep (Habib *et al* 2023). These stages are characterized by distinct physiological changes that are reflected in the PPG signal, such as heart rate slowing during deep sleep or respiratory irregularities during REM sleep. Emerging applications include detecting sleep disorders like OSA, where periodic drops in oxygen saturation and respiratory disruptions can be identified from PPG data. Additionally, wearable PPG devices can provide longitudinal sleep insights, supporting personalized health interventions and improving overall sleep quality (Sastimoglu *et al* 2025). Despite its promise, challenges such as motion artifacts and reduced signal quality during poor skin contact (Ren *et al* 2025) or low perfusion need to be addressed to enhance the reliability of PPG-based sleep analysis. Nevertheless, its widespread integration into wearable technologies makes it a valuable tool for improving sleep health and detecting related conditions.

Body posture significantly influences the frequency and severity of apneas and hypopneas during sleep (Oksenberg *et al* 2000, Leppänen *et al* 2016). Supine events are generally more severe, with longer durations, deeper desaturations, louder snoring, and stronger cardiovascular responses (Oksenberg *et al* 2000). These posture-related variations may impact the PPG waveform, but this has not been investigated in detail. In addition to distinguishing between apneas vs hypopneas, we wanted to see if the PPG signal is sensitive enough to distinguish between these two respiratory events occurring in different body sleep postures. Since supine apneas are, in general, more severe when occurring in the supine vs the lateral posture, we expected that these severity differences would be expressed by the PPG signal.

The primary objective of this study is to investigate how apnea and hypopnea events uniquely affect PPG waveform patterns. This is primarily because OSA is a syndrome affecting millions of adults worldwide, and making its detection and diagnosis more accessible could significantly improve the well-being of many people (Benjafield *et al* 2019). Specifically, we present a quantitative analysis of PPG signals during these events occurring in sleep stage N2. Additionally, we examine characteristic differences in PPG waveforms between events occurring in supine versus lateral positions and assess how these patterns vary over the course of the night.

## 2. Methods

### 2.1. The Multi-Ethnic Study of Atherosclerosis (MESA)

The MESA study investigates risk factors for subclinical CVD in adults without evident CVD, focusing on a diverse, community-based cohort (Chen *et al* 2015). Between 2010 and 2013, 2237 participants in the MESA Exam 5 completed type 2 polysomnograms (PSGs). All participants completed a standardized questionnaire to assess their medical, sleep, and lifestyle habits. The study received institutional review board approval, with all participants providing informed consent. Modified: PSG data, totaling over 2000 records, are accessible via the National Sleep Research Resource (NSRR) website (Zhang *et al* 2018). Sleep data were collected using a 15-channel Compumedics monitor, incorporating EEG, EOG, EMG, and inductance bands; and a nasal cannula for the assessment of the airflow and finger pulse oximetry, sampled at 1 Hz; the PPG signal was sampled at 256 Hz; position signal was recorded using a built-in sensor on the position indicator board, attached to the monitoring unit vest, with a sampling frequency of 32 Hz. The position states are defined as: 0 = right, 1 = back, 2 = left, 3 = prone, and 4 = upright. Respiratory events were marked by significant amplitude reductions in nasal pressure exceeding 30% for hypopneas and 90% for apneas for at least 10 s. AHI calculation included all apneas and hypopneas associated with >3% desaturation or arousal. The MESA dataset was downloaded from the National Sleep Research Resource—NSRR (sleepdata.org). This open dataset is used under an Institutional Review Board and Human Subjects Protection from the Technion-IIT Rappaport Faculty of Medicine (62-2019).

### 2.2. The patient population selection

**Figure 1.**
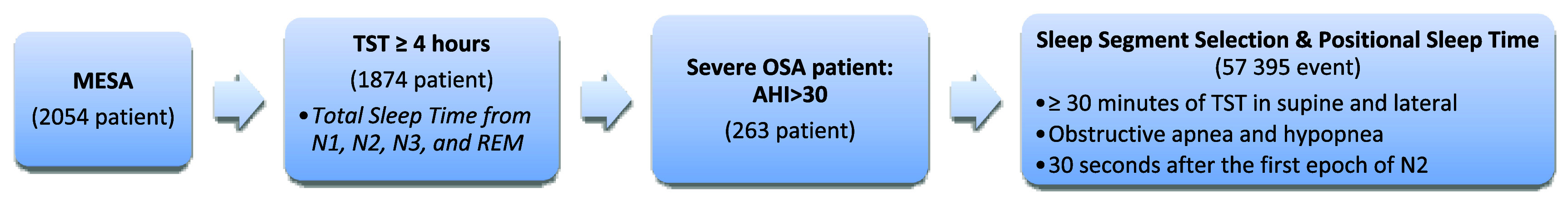
Workflow for event annotation and subject selection using inclusion and exclusion criteria.

We applied the following inclusion criteria for patient selection (see figure 1): *total sleep time (TST), calculated as the sum of N1, N2, N3, and REM sleep stages:* Patients with OSA whose TST was ⩾4 h. *Sleep segment selection*: obstructive apnea and hypopnea events were selected starting 30 s after the first epoch of N2 and continuing until the final sleep stage epoch of the TST. *Positional Sleep Time*: Patients with severe OSA who spent at least 30 min of TST in both the supine and lateral positions. Using the annotations provided by NSRR, apnea, hypopnea, and position data were extracted. Apneas were identified when the thermocouple signal was flat or nearly flat for more than 10 s (Chen *et al* 2015). Hypopneas were scored when the amplitude of either the combined abdominal and thoracic inductance signals or the nasal pressure flow signal decreased by at least 30% for 10 s or longer. Events were classified as either ‘central’ or ‘obstructive’ based on the presence or absence of respiratory effort. The AHI was calculated as the average number of apneas and hypopneas per hour of sleep; each event of apnea and hypopnea was selected regardless of desaturation or arousal. Sleep-disordered breathing (SDB) was defined as an AHI of ⩾5 events/hour, and further categorized as: mild (AHI 5–14), moderate (AHI 15–29), and severe (AHI ⩾ 30) (Chen *et al* 2015). For this study, we chose only severe OSA patients. The start and end points of the annotated apnea and hypopnea events determined the duration of each episode, the severe OSA with an AHI equal or above 30 events/h.

### 2.3. Patients selection summary

From the initial dataset of 2054 patients, 1874 patients met the criteria of having a TST of ⩾4 h and spending at least 30 min in both the supine and lateral positions. Among these 1874 patients, 979 were identified as having OSA, classified as follows: the cohort included 305 patients with mild OSA (AHI between 5 and 15), 411 patients with moderate OSA (AHI between 15 and 30), and 263 patients with severe OSA (AHI ⩾ 30), of whom 153 were male and 110 were female. The cohort included 305 patients with mild OSA (AHI between 5 and 15), 411 patients with moderate OSA (AHI between 15 and 30), and 263 patients with severe OSA (AHI ⩾ 30), of whom 153 were male and 110 were female. In our study, we concentrated all the analysis on the 263 severe OSA patients which allow us to have a high quantity of apnea and hypopneas in both supine and lateral posture across the night. Permission to use retrospective medical databases was granted following internal Institutional Review Board (IRB #62-2019).

The respiratory events of the 263 severe OSA patients were categorized into four groups: Lateral Apnea, Lateral Hypopnea, Supine Apnea, and Supine Hypopnea. The start and end points of the annotated apnea and hypopnea events determined the duration of each episode. Episodes with position changes during apnea or hypopnea events were excluded from further analysis.

### 2.4. Respiratory events classification according to PPG signal

**Figure 2.**
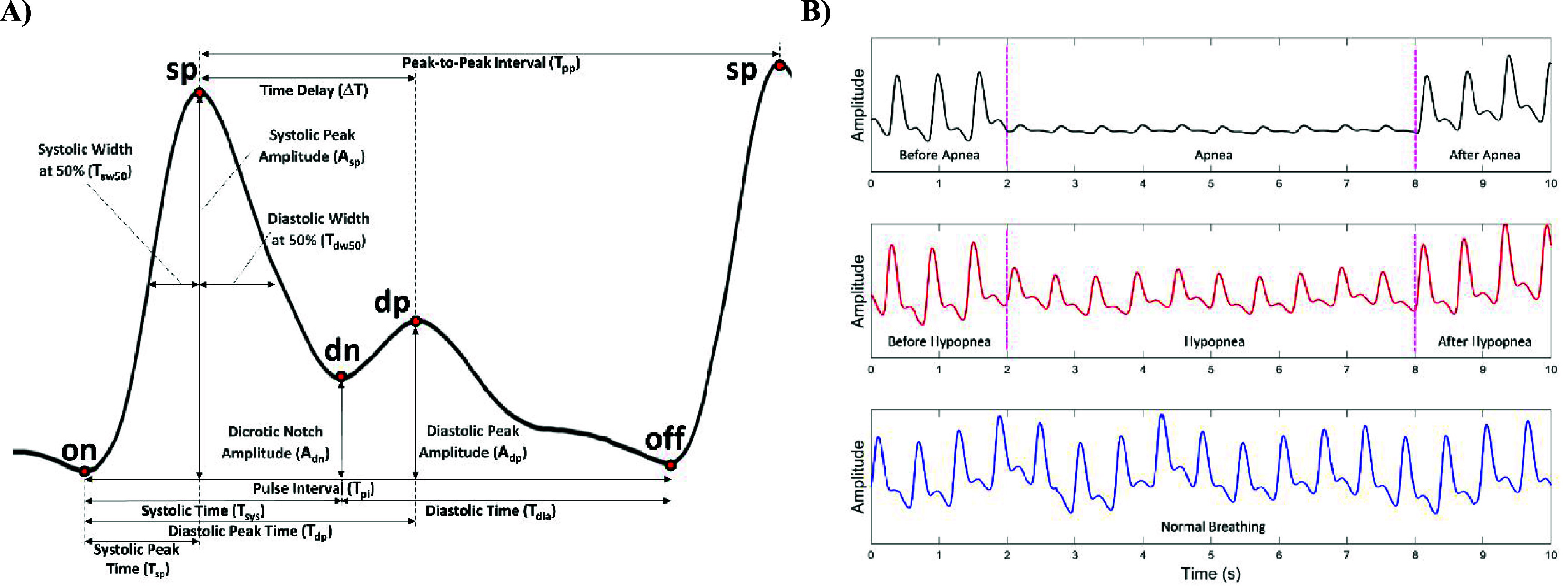
PPG characteristic waveform and the effect of apnea and hypopnea events on the waveform. (A) Fiducial points and characteristic biomarkers of the PPG signal, including peak-to-peak intervals, diastolic peak amplitude, and time delay. Adapted from Goda *et al* (2024). The Author(s). CC BY 4.0. (B) This figure illustrates the characteristic amplitude modulation of the PPG signal for Apnea, Hypopnea, and Normal Breathing. Adapted from Akbarian *et al* (2021). CC BY 4.0.

In 2024, Goda *et al* (2024) introduced a standardized pyPPG toolbox for PPG feature extraction (see figure 2 and tables 1–2). We extracted 74 features from the pyPPG toolbox and additionally 15 beat-rate variability (BRV) features from the PPG signal. During the training process, we identified the best 10 features for respiratory event classification. For respiratory events classification, we trained models for four binary classification tasks: **A.** Supine Apnea vs Lateral Apnea, **B**. Supine Hypopneas vs Lateral Hypopnea, **C.** Supine Apnea vs Supine Hypopnea, **D**. Lateral Apnea vs Lateral Hypopnea.

**Table 1.** The following table describes the names, definition, and units of the selected pyPPG features.

Name	Definition	Unit
*Ac/Aa*	Ratio of the *c*-point amplitude versus the *a*-point amplitude	(nu)
*Adn*	Dicrotic notch amplitude, the difference in amplitude between the pulse onset and dicrotic notch	(nu)
*Adp*	Diastolic peak amplitude, the difference in amplitude between the pulse onset and diastolic peak	(nu)
*Asp*	Systolic peak amplitude, the difference in amplitude between the pulse onset and systolic peak	(nu)
*IPR*	Instantaneous pulse rate, 60/Tpi	(%)
*Tb/Tpi*	Ratio of the *b*-point time versus the pulse interval	(nu)
*Tb-d*	*b*−*d* time, the time between the *b*-point and *d*-point	(s)
*Td*	*d*-point time, the time between the pulse onset and *d*-point	(s)
*Td/Tpi*	Ratio of the *e*-point time versus the pulse interval	(nu)
*Tdw10*	Diastolic width, the width at 10% of the systolic peak amplitude between the systolic peak and pulse offset	(s)
*Te*	*e*-point time, the time between the pulse onset and *e*-point	(s)
*Tf*	*f*-point time, the time between the pulse onset and *f*-point	(s)
*Tf/Tpi*	Ratio of the *f*-point time vs the pulse interval	(%)
*Tp1*	*p*1-point time, the time between the pulse onset and *p*1-point	(s)
*Tpp*	Peak-to-peak interval, the time between two consecutive systolic peaks	(s)
*Tpw25/Tpi*	Ratio of the pulse width at 25% of the systolic peak amplitude versus the pulse interval	(nu)
*Tpw75*	Pulse width, the sum of the systolic width and diastolic width at 75%	(s)
*Tpw75/Tpi*	Ratio of the pulse width at 75% of the systolic peak amplitude versus the pulse interval	(nu)
*TSys*	Systolic time, the time between the pulse onset and dicrotic notch	(s)
*Tv/Tpi*	Ratio of the *v*-point time versus the pulse interval	(nu)
*Tw*	*w*-point time, the time between the pulse onset and *w*-point	(s)
*Tw/Tpi*	Ratio of the *w*-point time vs the pulse interval	(%)

**Table 2.** The following table describes the names, definitions, and units of the selected BRV features.

Name	Definition	Unit
*IALS*	Inverse average length of segments (IALS)	(1/s)
*mean_dRR_*	The mean differences of successive beat-to-beat wave intervals	(s)
*RMSSD*	The RMSSD measure over a segment of the beat-to-beat time series	(ms)
*SD2*	The standard deviations from axis 2 and the length of the ellipse (long-term variability)	(ms)
*SDNN*	The standard deviation over beat-to-beat RR intervals (SDNN)	(ms)
*SEM*	Standard Error of the Mean (SEM) over a segment of peak-to-peak time series	(ms)

#### 2.5. Characteristics of breathing events across the night

For this analysis, we divided the sleep recording night into three thirds: beginning, middle and end also, we evaluated the results for the entire night. We first quantify the number of apneas and hypopnea events for each third of the night. Feature extraction was conducted across the entire length of apnea and hypopnea events.

*Terminology for sleep analysis:*

• ***Start***: The first sleep epoch of the total sleep period.
• ***TST duration***: The sleep time (N1 + N2 + N3 + REM sleep) from the first to the last sleep stage epoch.
• ***Beginning***: The interval from the first sleep stage epoch to the last sleep epoch of the first sleep third period.
• ***Middle***: The interval from the first sleep epoch of the second third to the last sleep epoch of the second sleep third period.
• ***End***: The interval from the first sleep epoch of the last sleep third period to the last sleep stage epoch.
• ***Entire***: The full sleep time from the start to the complete TST period.

### 2.6. Selected cohort

In this study, we assess sleep-related breathing abnormalities (Apneas + Hypopneas) events of 263 patients with severe OSA. Table 3 shows demographic and sleep information, as well as characteristics of respiratory events in these OSA patients. We analyzed 57 395 respiratory events with an average of almost 200 events/patient. These severe OSA patients were old (69.63 ± 8.99), and 57.17% of them were men. The TST was 6.23 ± 1.07 h with a relatively low sleep efficiency of 57.37 ± 13.52%. Not surprisingly, N2 was the dominant sleep stage of the night.

**Table 3.** Demographic information, sleep data, and characteristics of respiratory events were evaluated in the 263 severe OSA patients from mesa.

Name	Values
Severe OSA patients, *N*	263
Total No. respiratory events	57 395
Mean No. respiratory events/patient	194.73
AHI (mean ± SD)	41.83 ± 10.97
Age, years (mean ± SD)	69.6 ± 9
Sex, % of men	57.17%
Total recording time (mean ± SD), h	10.52 ± 1.36
Total sleep time (TST) (mean ± SD), h	6.23 ± 1.07
Sleep efficiency, % ± SD	57.37 ± 13.52%
N1, h (% of TST)	1.14 ± 0.66 (18.76%)
N2, h (% of TST)	3.54 ± 0.99 (56.64%)
N3, h (% of TST)	0.50 ± 0.48 (7.86%)
REM sleep, h (% of TST)	1.05 ± 0.50 (16.74%)
Apnea length, s (mean ± SD) *N* = 13 323	28.77 ± 11.34
Hypopnea length, s (mean ± SD) *N* = 43 627	18.37 ± 19.15
Supine apnea length, s (mean ± SD) *N* = 8755	28.9 ± 11.2
Supine hypopnea length, s (mean ± SD) *N* = 18 310	18.5 ± 28.4
Lateral apnea length, s (mean ± SD) *N* = 5013	28.5 ± 11.5
Lateral hypopnea length, s (mean ± SD) *N* = 25 317	18.3 ± 7.2
Supine apnea length N2, s (mean ± SD) *N* = 3946	29.9 ± 10.2
Supine hypopnea length N2, s (mean ± SD) *N* = 11 435	18.7 ± 33.9
Lateral apnea length N2, s (mean ± SD) *N* = 1889	29.1 ± 10.1
Lateral hypopneas length N2, s (mean ± SD) *N* = 13 760	18.6 ± 7.1

### 2.7. Statistical analysis

We performed statistical analysis by computing the mean, standard deviation, median, and Q1–Q3 range. To visualize the distribution of the features and event lengths, we utilized violin plots. Outliers were excluded from the violin plot by retaining data within the 1st and 99th percentiles. For significance testing, we first assessed normality using the Kolmogorov–Smirnov test. To evaluate differences between classified subgroups, we applied the Mann–Whitney U test and used a significance level of *p*-value at 0.05. Additionally, to examine significant differences and trends across night stages of TST (beginning, middle, end, and total), we employed the Kruskal–Wallis test.

Figure S3 shows that the duration of apneas is significantly longer than hypopneas in the three segments of the night, independently of the body posture where these events occur. In addition, this figure shows that the length of apneas and hypopneas in both lateral and supine posture did not change importantly across the night.

### 2.8. Model training and feature selection

To ensure patient-level separation and prevent data leakage, the dataset was split using GroupShuffleSplit, assigning 80% of the data to the training set and 20% to the test set, stratified by patient identifiers. A logistic regression model with recursive feature elimination was employed to perform embedded feature selection. Although other classifiers such as XGBoost and random forest were evaluated, they did not yield improved performance and incurred significantly higher computational costs.

The training and evaluation procedure was repeated 100 times, each with a different random train-test split, to enhance robustness and identify consistently informative features. Standardization was applied to input features prior to model training to normalize feature scales. For each iteration, we recorded the training and test partitions, predicted class labels and probabilities, selected features, and class distributions. Feature selection frequencies across iterations were aggregated to identify stable predictors.

**Figure 3.**
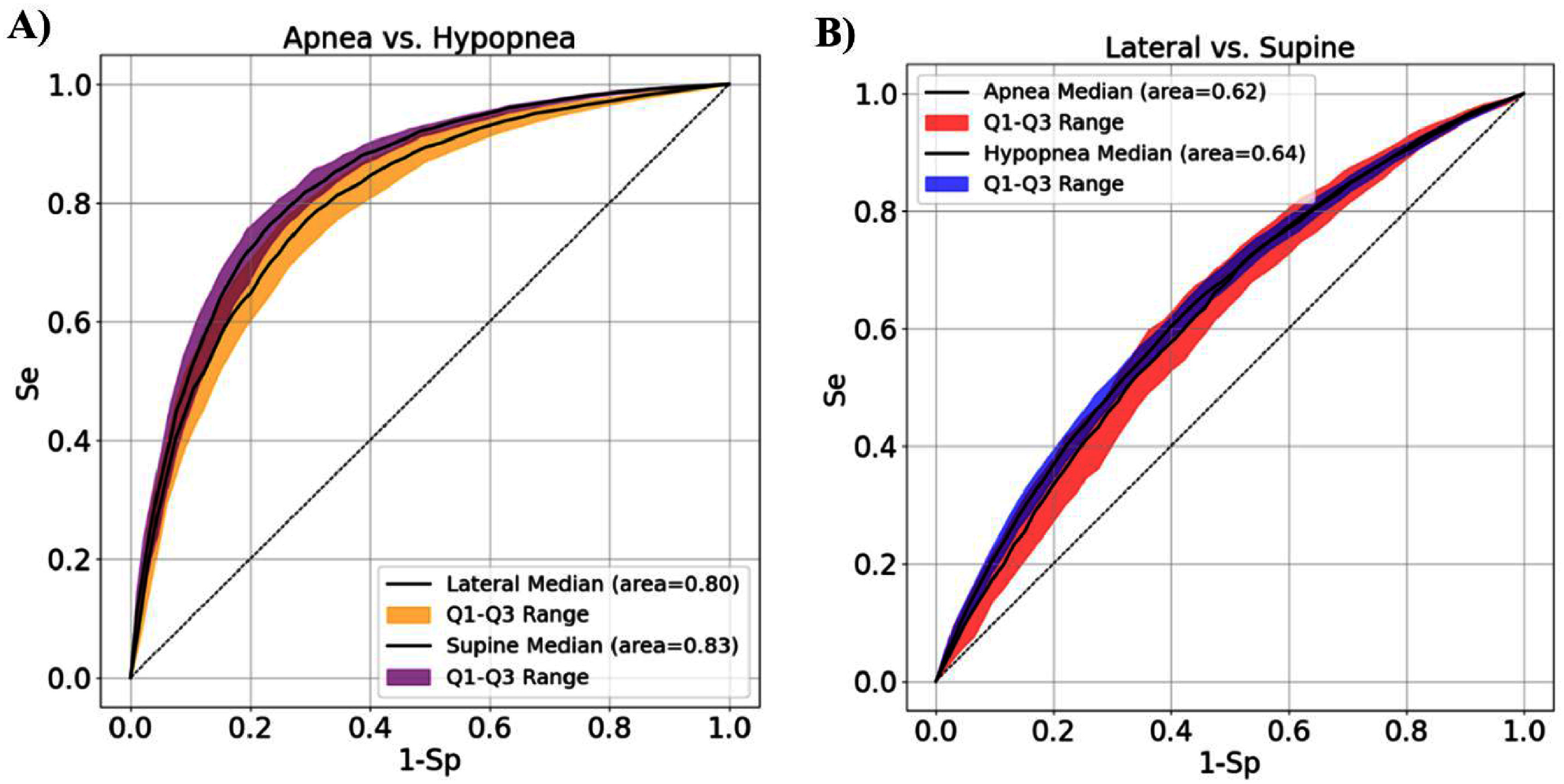
This figure illustrates the ROC curves for (A) Apnea vs Hypopnea, (B) Lateral vs Supine. While the task of lateral versus supine hypopnea/apnea classification shows lower predictive performance, the prediction accuracy for detecting apneas vs hypopneas is notably higher.

While classification performance for body position was relatively lower, the model achieved high accuracy in distinguishing Apnea from Hypopnea events across both supine and lateral positions (see figure 3). This pipeline was designed to support the development of reproducible and generalizable models, with a particular focus on identifying robust feature subsets for binary classification tasks.

## 3. Results

Using PPG signal characteristics, we classified two types of tasks: (figure 3(A)) Apneas and Hypopneas according to Lateral vs Supine position, and (figure 3(B)) Apnea vs Hypopnea differentiation. The results indicate that distinguish Apneas and Hypopneas according to both body postures (Lateral vs Supine) exhibits lower predictive performance compared to respiratory events detection (Apnea vs Hypopnea), where the model achieves greater accuracy.

Evaluation of respiratory events of all PSG recordings revealed that apneas were significantly longer than hypopneas, regardless of body posture. This pattern held for both lateral and supine events occurring during N2 sleep. Interestingly, supine apneas were similar in length to lateral apneas, and the same was true for hypopneas. This trend persisted even when restricting the analysis to N2 sleep. Notably, the average difference in length between apneas and hypopneas across all conditions was approximately 10 s, representing a significant disparity (see table 3). The evaluation of the duration of the respiratory events was also done to have a sense of the severity of apneas vs hypopneas.

### Discriminative PPG signals characteristics of respiratory events throughout the night

**Figure 4.**
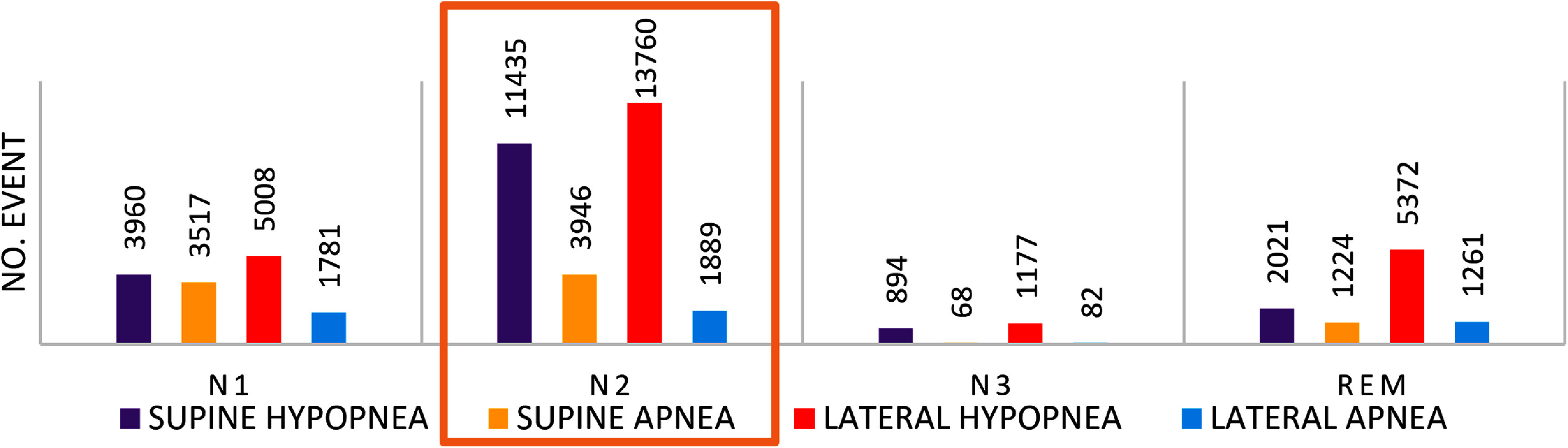
This figure shows the distribution of respiratory events across sleep stages. Training and validation datasets consisted solely of the N2 sleep stage.

Figure 4 shows the distribution of respiratory events across sleep stages. Note the small number of breathing events during N3 and that lateral hypopneas are the most common breathing event in all sleep stages. The largest number of respiratory events was found during N2. Most of the respiratory events, mainly hypopneas, occurred during N2, the dominant sleep stage. These previous results are a surprise. Since during this sleep stage we have enough respiratory events during the entire night, we assessed the analysis of apneas and hypopneas with PPG only during N2 in both lateral and supine posture and during the three thirds of the night to evaluate the possible changes in respiratory event duration across the night.

**Figure 5.**
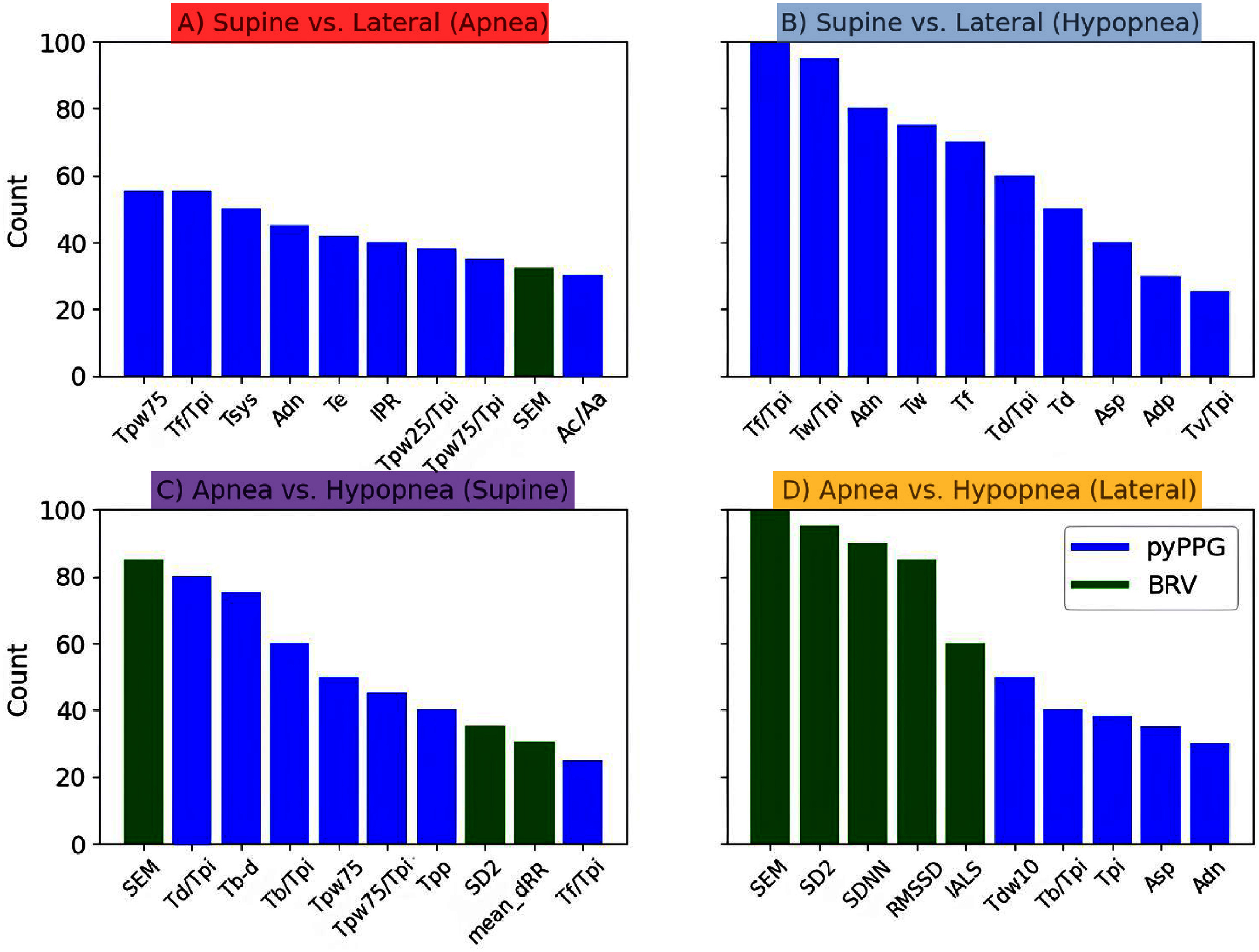
This figure presents the top ten features based on the maximum relevance minimum redundancy using 100 different cases. In the comparison of respiratory events classification between supine vs lateral positions for both Apnea (A) and Hypopnea (B), pyPPG based time-related features (blue columns) are predominant. In the comparison of Apnea vs Hypopnea within the Supine (C) and Lateral (D) positions, beat-rate variability (BRV) features (green columns) play also a dominant role.

The selected features (see figures 5 and S4) play a crucial role in classification performance. Overall, we observed that time-related features of pulse intervals (e.g. the time between fiducial points) or selected events (such as beat rate variability features) are generally more important than other feature types. Furthermore, BRV-type features showed weak discriminative power for classifying apneas and hypopneas according body sleep posture (see figures 5(A) and (B)). Based on our analysis, we classified the results into three groups according to the PPG signal as follows:

### Discriminative PPG signals characteristics for apneas vs hypopneas

Based on the PPG features, our machine learning model for classifying apnea versus hypopnea achieved AUROCs of 0.80 and 0.83 for lateral and supine events, respectively (see figure 3(A)). Although the difference in amplitude modulation between apneas and hypopneas is well-known (Akbarian *et al* 2021), our analysis found that time-related features were the most dominant (see figures 3(C) and (D)). The model’s discriminative performance remained consistent across the entire night. It is important to note that the apnea and hypopnea event durations were not included in the model training.

### Discriminative PPG signals characteristics between apneas and hypopneas according to sleep body postures

Based on the PPG features, our machine learning model for classifying lateral vs supine positions (Supine Apnea vs Lateral Apnea, and Supine Hypopneas vs Lateral Hypopnea) achieved AUROCs below 0.65 for both apnea and hypopnea events (see figure 3(B)), with consistent performance throughout the entire night. Additionally, we did not observe significant discriminative differences in event duration based on body position, either within specific sleep stages or across the entire recording.

Figure S1 highlights the feature differences among Lateral Apnea, Lateral Hypopnea, Supine Apnea, and Supine Hypopnea, which play a crucial role in machine learning-based classification. The figure clearly shows that the most significant differences are between apnea and hypopnea features, while the distinctions between supine and lateral positions are relatively minor. As shown in figure S2, most of the events are Hypopnea events, accounting for over 75% of all respiratory events. While Apnea events are predominantly observed in the supine position, Hypopnea events are more common in the lateral position.

**Figure 6.**
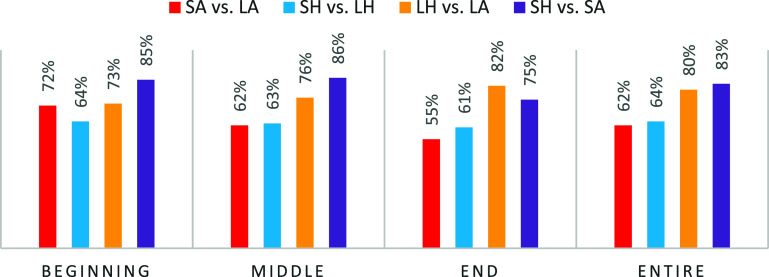
This figure depicts the area under the receiver operating characteristic (AUROC) results for the four comparisons of respiratory events during the three thirds of the sleep night: the beginning, middle, end, and entire duration. The event classifications include LA (Lateral Apneas), LH (Lateral Hypopneas), SA (Supine Apneas), and SH (Supine Hypopneas).

Table 4 presents the overall classification performance, including AUROC, precision, sensitivity, specificity, and *F*1 score. Figure 6 illustrates the area under the receiver operating characteristic (AUROC) values obtained from four distinct comparisons of respiratory events across the three different segments of the sleep period: the beginning, middle, end, and also, we assessed the AUROC for the entire night. These comparisons aim to evaluate the model’s ability to distinguish respiratory events during specific thirds of the night, as well as its overall performance throughout the full sleep duration. The AUROC values provide insight into how predictive performance varies depending on the temporal distribution of events, highlighting potential differences in event characteristics or detection accuracy at different times during sleep. The Supine Apneas vs Supine Hypopneas showed the best performance on the beginning, middle and for the entire night with a small decrease in the end portion of the night.

**Table 4.** Classification performance summary.

	Supine		Lateral		Apnea		Hypopnea	
	Apnea vs Hypopnea		Apnea vs Hypopnea		Supine vs Lateral		Supine vs Lateral	
	*Mean ± STD*	*Median* (*Q1–Q3*)	*Mean ± STD*	*Median* (*Q1–Q3*)	*Mean ± STD*	*Median* (*Q1–Q3*)	*Mean ± STD*	*Median* (*Q1–Q3*)
AUROC	0.83 ± 0.04	0.83 (0.81–0.86)	0.80 ± 0.05	0.81(0.76–0.84)	0.62 ± 0.06	0.63 (0.59–0.66)	0.64 ± 0.04	0.64 (0.61–0.67)
Precision	0.84 ± 0.05	0.84 (0.81–0.87)	0.89 ± 0.04	0.90(0.87–0.92)	0.73 ± 0.08	0.73 (0.68–0.78)	0.59 ± 0.06	0.60 (0.55–0.64)
Sensitivity	0.92 ± 0.02	0.92 (0.91–0.94)	0.98 ± 0.01	0.98(0.97–0.99)	0.87 ± 0.07	0.88 (0.83–0.92)	0.47 ± 0.09	0.49 (0.42–0.54)
Specificity	0.50 ± 0.09	0.50 (0.44–0.56)	0.14 ± 0.07	0.13(0.09–0.19)	0.26 ± 0.10	0.26 (0.18–0.32)	0.72 ± 0.06	0.72 (0.69–0.76)
*F*1	0.88 ± 0.02	0.88 (0.86–0.90)	0.93 ± 0.02	0.94(0.93–0.95)	0.78 ± 0.05	0.79 (0.75–0.82)	0.52 ± 0.07	0.53 (0.49–0.57)

Our trained models have distinct trends throughout the night. The accuracy classification of the PPG signal for assessing Lateral Hypopnea vs Supine Hypopnea remains similar across the night. In contrast, the accuracy classification of the PPG signal for the distinction of Lateral Apnea vs Supine Apnea decreases toward the end of the night. The accuracy classification of the PPG signal for assessing the differences between Supine Apnea vs Supine Hypopnea shows an initial increase followed by a drop, while the accuracy classification of the PPG signal for Lateral Apnea vs Lateral Hypopnea increases steadily throughout the night. The decrease accuracy of the PPG signal to differentiate between Lateral Apnea vs Supine Apnea, could be related to the fact that in the last part of the night, REM sleep is dominant, and perhaps during this sleep stage, where the respiratory events are in general more severe (longer and with deeper desaturations) it harder for this PPG signal to be accurate in the distinction between these two respiratory events which are both longer. In other words, the possible similar severity of these breathing events during REM sleep, could be the factor that affect the accuracy of the PPG signal to distinguish between these two breathing events. To investigate if this explanation is correct, a comparison between the detection accuracy for the differentiation between apneas vs hypopneas of the PPG signal, between REM sleep vs NREM sleep is required and could be of interest.

## 4. Discussion

This study analyzed PPG signals during apneas and hypopneas in 263 patients with severe OSA, using data from nocturnal PSG recordings during sleep stage N2. We found that PPG features significantly differed between apneas and hypopneas, regardless of the body posture where these respiratory events occurred. Using features extracted with the recent pyPPG toolbox, our machine learning model achieved AUROCs of 0.80 and 0.84 for classifying lateral and supine events, respectively. These results demonstrate the potential of PPG for accurate, posture-independent detection of SDB, supporting its use in wearable, non-invasive diagnostics. The ability of the PPG signal to distinguish between apneas and hypopneas is particularly noteworthy. Apnea events are typically associated with more pronounced physiological disturbances than hypopneas, including longer durations, deeper oxygen desaturations, greater heart rate variability (bradycardia/tachycardia), stronger arousals, and louder snoring. These heightened responses likely result in more substantial blood volume changes in the microvascular bed of the finger, which are captured by the PPG signal. This physiological contrast may underlie the signal’s capacity to effectively differentiate between the two event types. Recently, (Luukinen *et al* 2024) studied the variation of the PPG responses to arousal and found that the magnitude of the vasoconstriction (expressed by the PPG) was larger if the respiratory event was associated with desaturation and the magnitude of the heart rate increase was larger following respiratory arousals vs spontaneous arousals, and larger after those caused by apneas vs hypopneas, showing also that the severity of the respiratory events are captured by the PPG signal responses.

PPG signals display distinct patterns during apnea and hypopnea events, reflecting the differing degrees of airway obstruction and their impact on autonomic function. During apneas, where breathing stops completely or nearly completely, the PPG waveform typically shows a marked reduction in pulse amplitude, often followed by a rebound increase due to a surge in sympathetic activity triggered by arousal. This is mirrored in heart rate variability, with bradycardia during the event and tachycardia immediately afterward. In contrast, hypopneas involve only partial airway obstruction, resulting in milder oxygen desaturation and subtler changes in the PPG waveform, including less pronounced heart rate shifts. Notably, when distinguishing between apneas and hypopneas within the same posture (Panels (C) and (D) of figure 5), BRV features (green bars) emerge as the most influential. This suggests that autonomic modulation, as reflected by BRV, plays a more significant role in differentiating event types than in detecting positional effects.

The capability of the PPG signal characteristics to differentiate breathing events (apneas and hypopneas) according to the body posture where they occur remained unclear. It has been known for already many years that respiratory events occurring in the supine posture are more severe than those occurring in the lateral position (Oksenberg *et al* 2000). The breathing events in the supine posture are in general longer, cause deeper desaturations, lead to higher Brady/tachycardia changes, ended with louder snoring sounds and are associated with longer and more severe arousals. Based on the PPG’s signal effectiveness in distinguishing between apneas vs hypopneas, owing most probably on the differences in severity of these respiratory events, we might have expected also from the PPG signal to differentiate respiratory events based on body posture were those breathing events occur. However, the results suggest that the PPG signal’s capability for posture-based differentiation of respiratory events is insufficient. This raises questions about the reason for these somehow unexpected results. We propose that the length of the respiratory events may explain at least partially these findings. Contrary to previous studies showing that respiratory abnormalities are longer in the supine position than in the lateral position (Leppänen *et al* 2016, Leppänen *et al* 2017, Kulkas *et al* 2017, Oksenberg *et al* 2000), the results of this study in 263 severe OSA patients and after analyzed thousands of apneas and hypopneas during N2, we show that apneas and hypopneas have similar durations in the supine and lateral posture with only a marginal difference. Most, but not all the previous studies showing an increase in the length of supine apneas vs lateral apneas have been assessed in a smaller number of OSA patients and more importantly in reduced number of apneas and hypopneas (Oksenberg *et al* 2000). However, since the previous studies show consistently that respiratory events occurring in the supine posture are longer that those occurring in the lateral posture, more studies assessing specifically this topic and using many OSA patients assessing all three degrees of OSA severity and with a large number of respiratory events are needed. The results showed that, regardless of the time of night, PPG signals were more effective at distinguishing between apneas and hypopneas than at identifying differences in the same respiratory event type based on body posture.

To normalize the data, we assess the number and length of these respiratory events in the beginning, middle and end of the sleep period but only during N2. As observed in figure S3 in the supplementary data, the lengths of apneas are consistently larger that hypopneas during all segments of the sleep period. However, contrary to several studies showing an increase in the length of the apneas toward the end of the night (Lavie *et al* 1981, Charbonneau *et al* 1994, Montserrat *et al* 1996, Oksenberg *et al* 2001) in our study, we could not replicate these previous results. That is, we did not observe an increase in the length of apnea or hypopneas events toward the end of the night.

In most of previous studies showing that the length of respiratory events increases toward the morning hours were performed in a significantly small number of OSA patients and a small number of respiratory events, less than thousand events (Lavie *et al* 1981, Charbonneau *et al* 1994, Montserrat *et al* 1996, Oksenberg *et al* 2001). In the study of (Lavie *et al* 1981), they analyzed data of 8 OSA patients with more than 200 apnea/night during sleep stage 2 (N2). In the (Charbonneau *et al* 1994) study, they reviewed PSG studies from 66 OSA patients with an AHI > 40, dividing bed time into equal quartiles. The authors found that the severity of apnea increased as the night progressed due to lengthening of mean apnea duration (mainly in NREM), increased proportion of REM sleep, and in the most severe patients, also an increase in AHI. In the study of (Montserrat *et al* 1996), they assessed apnea duration, SaO2, and inspiratory effort during apneas at the start and end of the night in Stage 2 (N2) sleep in 7 male with severe OSA. In the study of (Oksenberg *et al* 2001) PSG of 30 patients with severe OSA who had at least 30 apneic episodes in the lateral position and 30 in the supine position during Stage 2 (N2) sleep, in the early, middle and late sleep periods were analyzed. Although the present results should be corroborated in future studies, our results suggest that to evaluate whether the length of respiratory events change across the night, it is imperative to assess a large quantity of these respiratory events in a sizable amount of OSA patients to obtain trustworthy results. Similarly, our results on the effect of body posture on the length of respiratory events, was done in a considerable number of individuals and assessing a large number of respiratory events to obtain confident results. Nevertheless, since the results of these previous studies found consistently that respiratory events increase in length toward the morning, and also that the length of respiratory events occurring in the supine posture are larger than those occurring in the lateral position, contrary to the results of the present study, a corroboration of the results of the present study is imperative.

**Limitations**

Our study has several limitations that should be acknowledged. First, we focused specifically on evaluating the potential of the PPG signal to distinguish between the two primary breathing abnormalities associated with OSA, and to explore whether PPG can further differentiate apneas and hypopneas based on sleep posture. While this focus allowed for a targeted analysis, the clinical utility of PPG would be better demonstrated by also including the detection of normal breathing events, an important aspect that falls outside the scope of the current study.

Second, the analysis was conducted using data from a single database. Evaluating our approach on an independent dataset could have further strengthened the validity of our findings. To improve generalizability, future studies should replicate these analyses using at least one additional, large, and diverse dataset, preferably one that includes a wider age range, as our cohort had a relatively high mean age (69.63 ± 8.99 years).

Lastly, our analysis was limited to patients with severe OSA and focused exclusively on sleep stage N2 and obstructive apnea and hypopnea events. Future studies should examine PPG data across all sleep stages, include patients with varying levels of OSA severity, and incorporate a more detailed event classification that also considers mixed and central apneas. This would help ensure broader applicability and provide a more comprehensive understanding of PPG signal dynamics.

## 5. Conclusions

The results of this study demonstrate that PPG signal characteristics differ significantly between apneas and hypopneas during overnight PSG recordings in severe OSA patients during sleep stage N2, allowing for accurate differentiation between these two event types. However, the PPG signal showed limited ability to distinguish between apneas or hypopneas based on body posture (supine vs lateral). A plausible explanation for this could be the similar durations of events across postures, suggesting comparable severity and, thus, limited physiological contrast detectable by PPG. Importantly, our findings indicate that PPG signals can reliably differentiate apneas from hypopneas across all segments of the night, regardless of body position. We also observed that event durations remained stable throughout the night, with apneas consistently lasting longer than hypopneas. While these results are promising, further validation using additional large PSG datasets—including populations with a broader age range—is essential to confirm and generalize these findings.

## Data Availability

The data that support the findings of this study are openly available at the following URL/DOI: https://doi.org/10.25822/n7hq-c406 Supplementary Data available at http://doi.org/10.1088/1361-6579/ae3ef0/data1.
